# Tau-Targeted Immunization Impedes Progression of Neurofibrillary Histopathology in Aged P301L Tau Transgenic Mice

**DOI:** 10.1371/journal.pone.0026860

**Published:** 2011-12-08

**Authors:** Mian Bi, Arne Ittner, Yazi D. Ke, Jürgen Götz, Lars M. Ittner

**Affiliations:** 1 Laboratory for Translational Neurodegeneration, Brain and Mind Research Institute, The University of Sydney, Camperdown, New South Wales, Australia; 2 Alzheimer's and Parkinson's Disease Laboratory, Brain and Mind Research Institute, The University of Sydney, Camperdown, New South Wales, Australia; Federal University of Rio de Janeiro, Brazil

## Abstract

In Alzheimer's disease (AD) brains, the microtubule-associated protein tau and amyloid-β (Aβ) deposit as intracellular neurofibrillary tangles (NFTs) and extracellular plaques, respectively. Tau deposits are furthermore found in a significant number of frontotemporal dementia cases. These diseases are characterized by progressive neurodegeneration, the loss of intellectual capabilities and behavioral changes. Unfortunately, the currently available therapies are limited to symptomatic relief. While active immunization against Aβ has shown efficacy in both various AD mouse models and patients with AD, immunization against pathogenic tau has only recently been shown to prevent pathology in young tau transgenic mice. However, if translated to humans, diagnosis and treatment would be routinely done when symptoms are overt, meaning that the histopathological changes have already progressed. Therefore, we used active immunization to target pathogenic tau in 4, 8, and 18 months-old P301L tau transgenic pR5 mice that have an onset of NFT pathology at 6 months of age. In all age groups, NFT pathology was significantly reduced in treated compared to control pR5 mice. Similarly, phosphorylation of tau at pathological sites was reduced. In addition, increased astrocytosis was found in the oldest treated group. Taken together, our data suggests that tau-targeted immunization slows the progression of NFT pathology in mice, with practical implications for human patients.

## Introduction

Alzheimer's disease (AD) is a progressive neurodegenerative disease affecting more than 35 million people worldwide [1]. It is characterized by the loss of neurons that is associated with a progressive decline in cognitive functions. Frontotemporal dementia (FTD) describes a heterogeneous group of neurodegenerative disorders that are characterized by a broad spectrum of clinical symptoms including behavioural changes, language abnormalities and motor dysfunction [2]. FTD is the second most common form of dementia before the age of 65 [3]. Neither AD, FTD nor related dementias can be cured, and symptomatic treatment is very limited.

AD brains are characterized by the deposition of two hallmark proteins, the amyloid-β (Aβ) peptide and the microtubule-associated protein tau. Aβ is derived from the Aβ precursor protein (APP) and is the major constituent of plaques, while abnormally phosphorylated (hyperphosphorylated) tau gives rise to neurofibrillary tangles (NFTs) [4]. Aβ is closely associated with the onset of AD, but it is the tau pathology that correlates with its severity [5].

In FTD, tau pathology occurs in the absence of overt Aβ deposition [2]. In a subset of FTD, pathogenic mutations have been identified in the tau*-*encoding *MAPT* gene [6], [7]. Transgenic expression of mutant human tau has assisted in establishing robust mouse models that recapitulate features of human pathology [8], [9]. In addition, we and others recently showed that genetically reducing tau levels in tau-knockout mice prevented Aβ toxicity [10], [11]. Taken together, tau plays a central role in AD and FTD, making it an attractive drug target [1].Several approaches to therapeutically target tau have been devised [12]. However, while effective *in vitro*, only a limited number of agents have undergone testing in established transgenic models. To date only very few tau-directed drugs have progressed into human clinical trails [12]. Given the central role of tau in disease, there is a need for new therapeutic approaches targeting tau pathology.

Aβ-directed vaccination has raised the hope for an effective treatment for AD, and after initial setbacks in clinical trials, modified protocols for both active and passive immunization are back in clinical testing [13]. Although tau is an intracellular protein and therefore does not appear as an immunization target of first choice, active immunization of young mutant tau transgenic mice could prevent the formation of pathology [14], [15], [16]. In an effort to model the human condition at the time of diagnosis more closely, we here used active immunization against tau in aged tau transgenic mice with pre-existing pathology. This reduced both tau phosphorylation and NFT numbers, suggesting that therapeutic immunization against tau may prevent progression of disease.

## Results

### Tau-targeted immunization in pR5 mice

**Figure 1:**
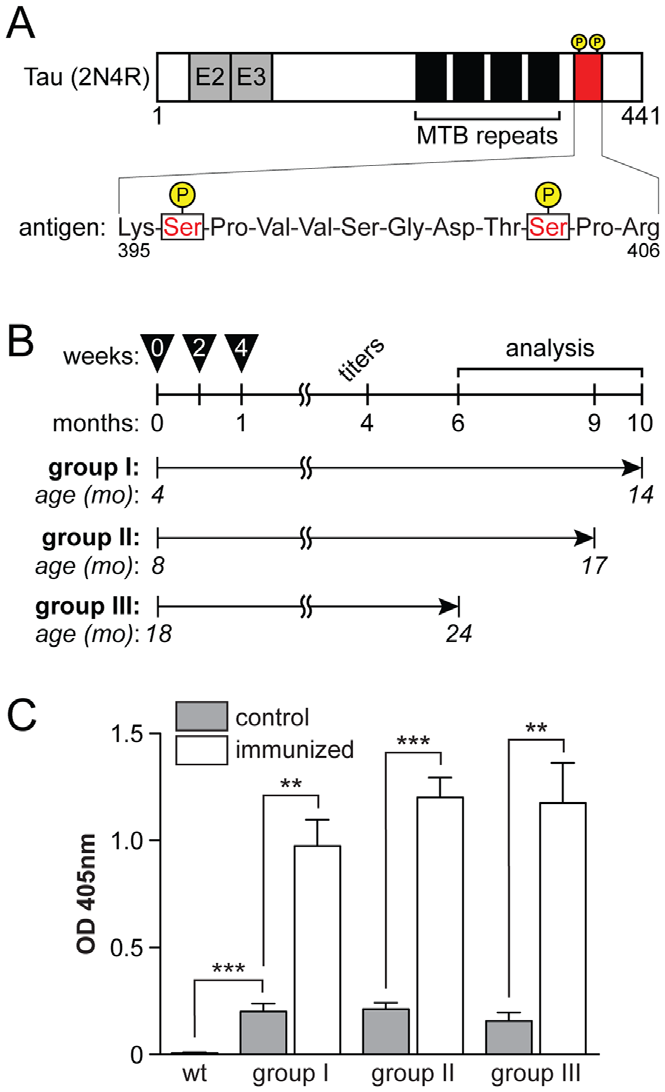
Immune response in vaccinated P301L tau expressing pR5 mice. (**A**) The longest human tau isoform (441 amino acids) includes two N-terminal repeats, E2 and E3, (2N) and four microtubule binding (MTB) repeats (4R). The antigen sequence (red) comprising the phosphorylation sites (P) Ser 396 and Ser 404 lies within the C-terminal tail region of tau. The sequence of the antigen is displayed at the bottom. (**B**) Three groups I–III of pR5 mice were sequentially immunized with KLH-linked antigen together with CFA or IFA at 0, 2 and 4 weeks. Four months after the first injection, antibody titers were determined in blood samples and eventually, mice were sacrificed for histopathological analysis at indicated times. (**C**) Antibody titers in serum of wild-type (wt), immunized and non-immunized (control) pR5 mice. Optic density (OD) at 405 nm of 1∶10 diluted sera shows high titers in immunized pR5 mice for all groups, low titer auto-antibodies in non-immunized pR5 controls, and no detectable antibodies in wt mice (**, *p*<0.001; ***, *p*<0.0001).

Phosphorylation of tau in the P301L mutant tau expressing pR5 line increases over time. While certain epitopes are highly phosphorylated early on, including Ser202 (AT8) and Ser235 (AT180), others show robust phosphorylation at later, more advanced, stages of pathology, including Ser422 and Ser396/S404 (PHF1) [17]. The latter phosphorylation sites have been associated with NFT formation [18]. Here, we used a twelve amino acid peptide of the human tau sequence (aa 395–406), comprising phosphorylated Ser396/Ser404 of the PHF-1 epitope (**Fig. 1** **A**), to immunize P301L mutant tau expressing pR5 mice of different ages. NFTs start to develop at 6 months of age in the amygdala of pR5 mice and their numbers progressively increase thereafter [18]. Therefore, the three test groups of at least eight pR5 mice each were four, eight and 18 months old at the start of the trial, resembling mice before the onset of (group I), with moderate (group II) or advanced (group III) NFT pathology (**Fig. 1** **B**). All mice were immunized with 100 µg of KLH-linked peptide as antigen together with CFA, followed by two additional injections of antigen and IFA two and four weeks later (**Fig. 1** **B**). Controls received equivalent amounts of KLH and CFA or IFA.

Four months after the initial immunization, peptide-treated pR5 mice developed robust titers of ≥1∶1000, suggesting a persistent antigen-specific immune response (**Fig. 1** **C**). Interestingly, antigen-specific titers were 1∶10 in all control pR5 mice, while the independent wild-type mice had no detectable titers. This suggests that transgenic expression of tau is associated with a low, but reproducible tau-specific immune response.

### Immunization reduces tau phosphorylation

Mice from groups I and II were sacrificed 9 to 10 months after the first antigen injection and processed for histopathological analysis. Mice from group III were sacrificed 6 months after the start of the treatment, due to their advance aged. Two control pR5 mice of group III had died by the time of analysis, and another control mouse lost up to 30% of its body weight, different from antigen-treated mice.

**Figure 2:**
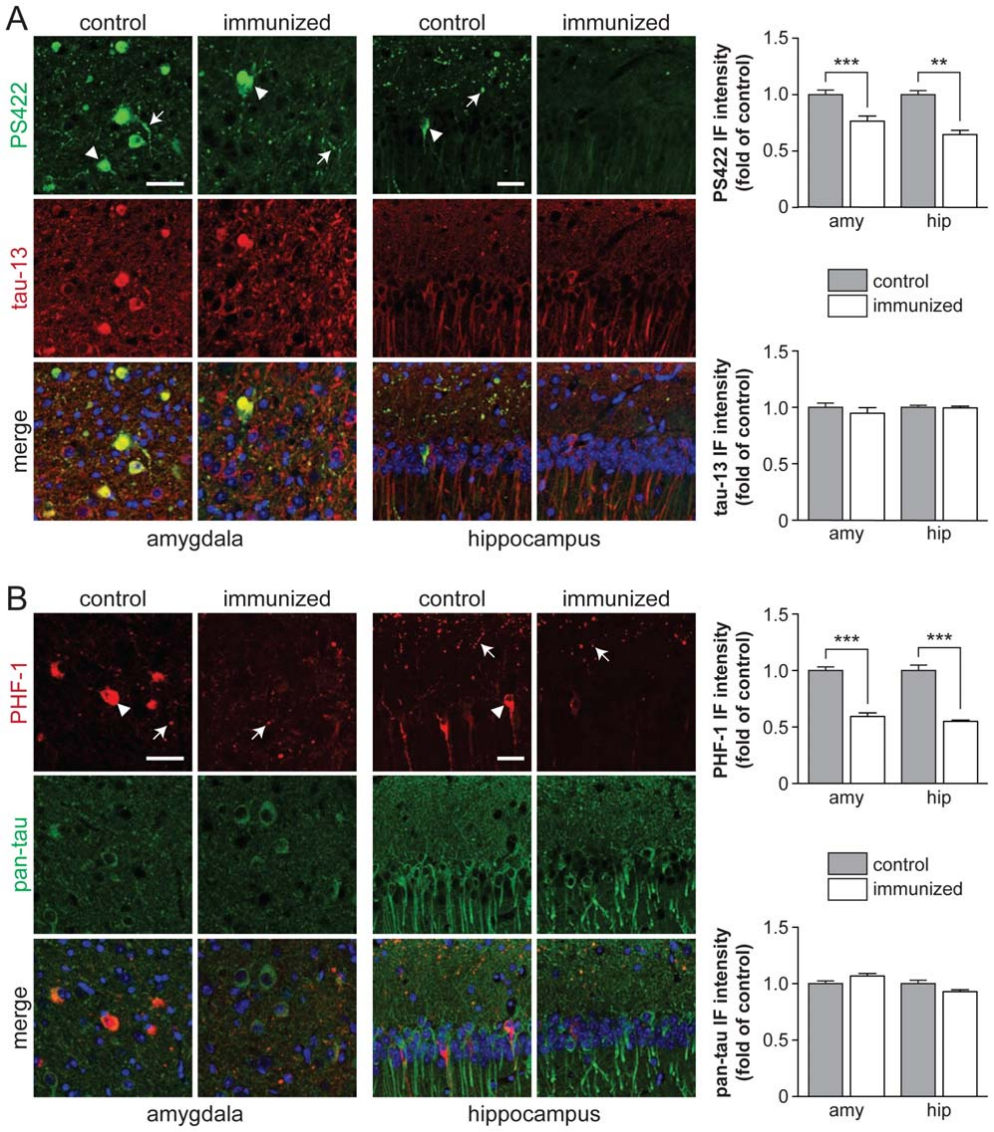
Tau-targeted immunization reduces tau phosphorylation in pR5 mice. (**A**) Immunohistochemistry (IHC) of coronal brain sections obtained from 17 months-old pR5 mice (group II) that have been immunized 9 months ago (immunized) or were control injected (control), stained with antibodies to total human tau (tau-13) and tau phosphorylated at Ser422 (PS422). Representative pictures from amygdala (left) and CA1 region of the hippocampus (right) were taken at the same exposure. Several neurons stained intensively with PS422 in these brain areas of control pR5 mice (arrowheads). Similarly, PS422 stained dystrophic neurites (arrows). In contrast, numbers of PS422-positive neurons in amygdala and hippocampus, as well as staining of dystrophic neurites in the hippocampus were strongly reduced in immunized pR5 mice, indicating reduced pathological phosphorylation of tau. Note that the staining pattern with tau-13 is comparable in control and immunized pR5 amygdala and hippocampus, suggesting similar levels of total tau. Merged pictures include nuclear DAPI staining. Scale bars, 50 µm. Graphs: Quantification of immunofluoresence intensity in total amygdala and CA1 region of the hippocampus shows significantly reduced PS422 staining in immunized compared to control pR5 mice, while tau-13 staining is similar (**, *p*<0.001; ***, *p*<0.0001). (**B**) Similar to PS422, an antibody to tau phosphorylated at Ser396/Ser404 (PHF-1) stained numerous neurons (arrowheads) and dystrophic neurites (arrows) in the amydala and hippocampus of control pR5 brains. Much less neurons in amygdala and hippocampus and dystrophic neurites in the hippocampus stained with PHF-1 in immunized pR5 mice, indicating reduced levels of tau phosphorylation at Ser396/Ser404. Staining for total tau (pan-tau; green) was comparable in control and immunized pR5 mice, suggesting again unchanged total tau levels. Scale bars, 50 µm. Graphs: Quantification of immunofluoresence intensity in total amygdala and CA1 region of the hippocampus shows significantly reduced PHF-1 staining in immunized compared to control pR5 mice, while pan-tau staining is similar (***, *p*<0.0001).

pR5 mice are characterized by a progressively increasing phosphorylation of tau at multiple sites [17]. As outlined above, phosphorylation of tau at the pathological sites Ser396/Ser404 (PHF-1 epitope) and Ser422 (PS422 epitope) occur late and is closely related to NFT formation in mice [18], [19]. To determine the degree of phosphorylation of tau at Ser396/Ser404 and Ser422, as well as total human tau expression, we used both phosphorylation- and human tau specific antibodies. Immunization had no overt effect on tau expression and distribution pattern, since both human and total tau specific antibody staining was similar in antigen-treated and control pR5 mice (**Fig. 2** **A and B**). Consistent with our previous studies in pR5 mice, we found intensive somatodendritic staining of neurons in hippocampus and amygdala of 17 months-old pR5 controls (group II) with antibodies specific to tau phosphorylated at Ser396/Ser404 (PHF-1) or Ser422 (PS422) (**Fig. 2** **A and B**). In addition, both PHF-1 and PS422 stain dystrophic neurites in the amygdala and the axonal layer of the hippocampus. In antigen-treated pR5 mice, however, only a few neurons stain with PHF-1 or PS422. Similarly, PHF-1 and PS422 staining of dystrophic neurites in the hippocampus of antigen-treated pR5 mice is low compared to controls. Hence, phosphorylation of tau at Ser396/Ser404 and Ser422 is reduced by tau-targeted immunization.

### Immunization slows progression of NFT pathology

**Figure 3:**
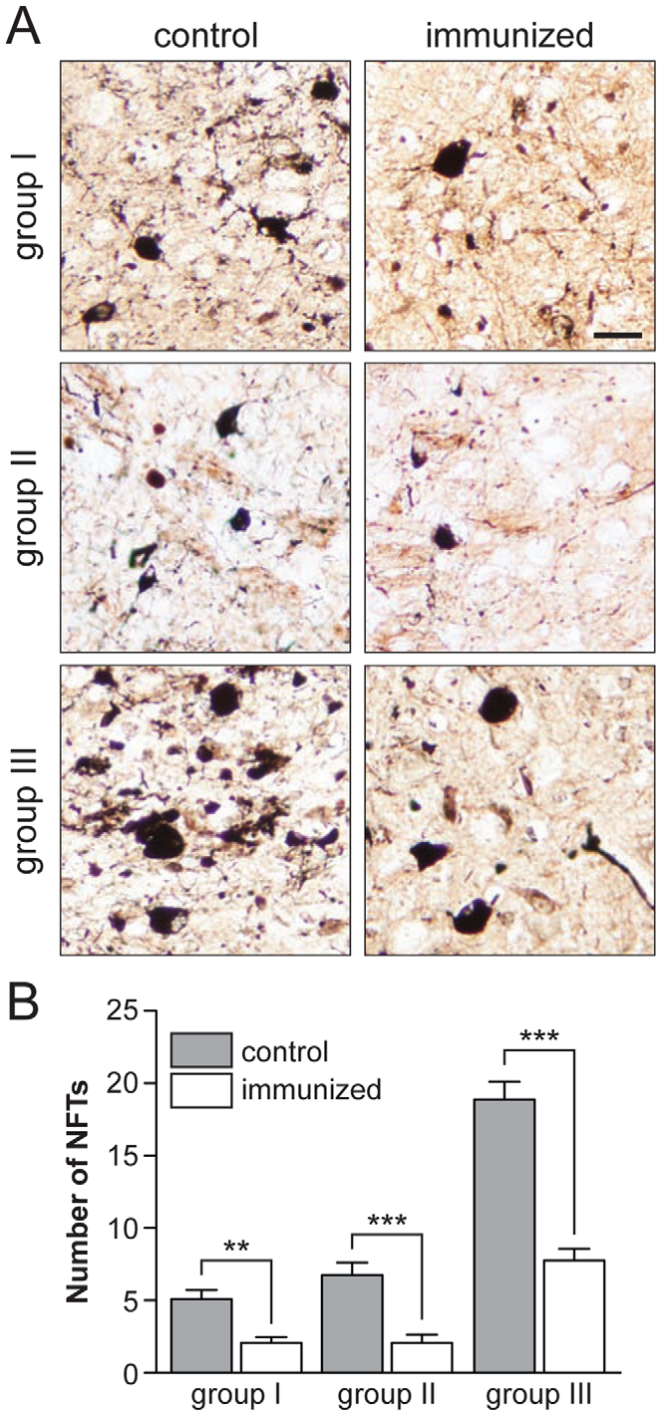
Tau-immunized pR5 mice have less NFTs. (**A**) Gallyas silver staining of coronal brain sections from 14 (group I), 17 (group II) and 24 (group III) months-old pR5 mice, respectively. Representative images of amygdalae of control pR5 brains of all three groups reveal numerous Gallyas silver-positive NFTs (‘flame shaped’), together with dystrophic neurites. In the brains of immunized pR5 mice, there are much less NFTs and dystrophic neurites stained, suggesting reduced NFT pathology. Scale bar, 25 µm. (**B**) Quantification of NFT numbers in serial sections from control and immunized pR5 mice stained with Gallays silver impregnation shows significantly reduced NFT numbers in immunized pR5 mice in all three treatment groups, compared to the respective controls. **, *p*<0.001 and ***, *p*<0.0001; Student *t*-test (controls vs. immunized); n = 12 for group I and II, and n = 8 for group III.

Similar to AD brains, numbers of NFTs progressively increase in pR5 mice after the onset of pathology [5], [20]. We used Gallyas silver impregnation to stain NFTs in pR5 paraffin sections, a method routinely used in AD post-mortem diagnostics (**Fig. 3** **A**). Numbers of NFTs in the amygdala were determined on serial sections, to determine progression of pathology as described before [18], [21]. In all three age groups, numbers of NFTs were significantly lower in antigen-treated pR5 compared to the respective controls (group I: *p*<0.001, groups II/III: *p*<0.0001) (**Fig. 3** **B**). For comparison, NFT numbers in control pR5 mice were in agreement with previous studies [17]. However, NFTs were present in all antigen-treated pR5 brains, although at lower number (**Fig. 3** **B**). In particular in groups I and II, numbers of NFTs were higher than what would have been expected if tau-targeted immunization were to prevent *de novo* formation of NFTs, suggestive of a slowed rather than halted progression of NFT pathology.

### Astrocytosis in aged immunized pR5 mice

**Figure 4:**
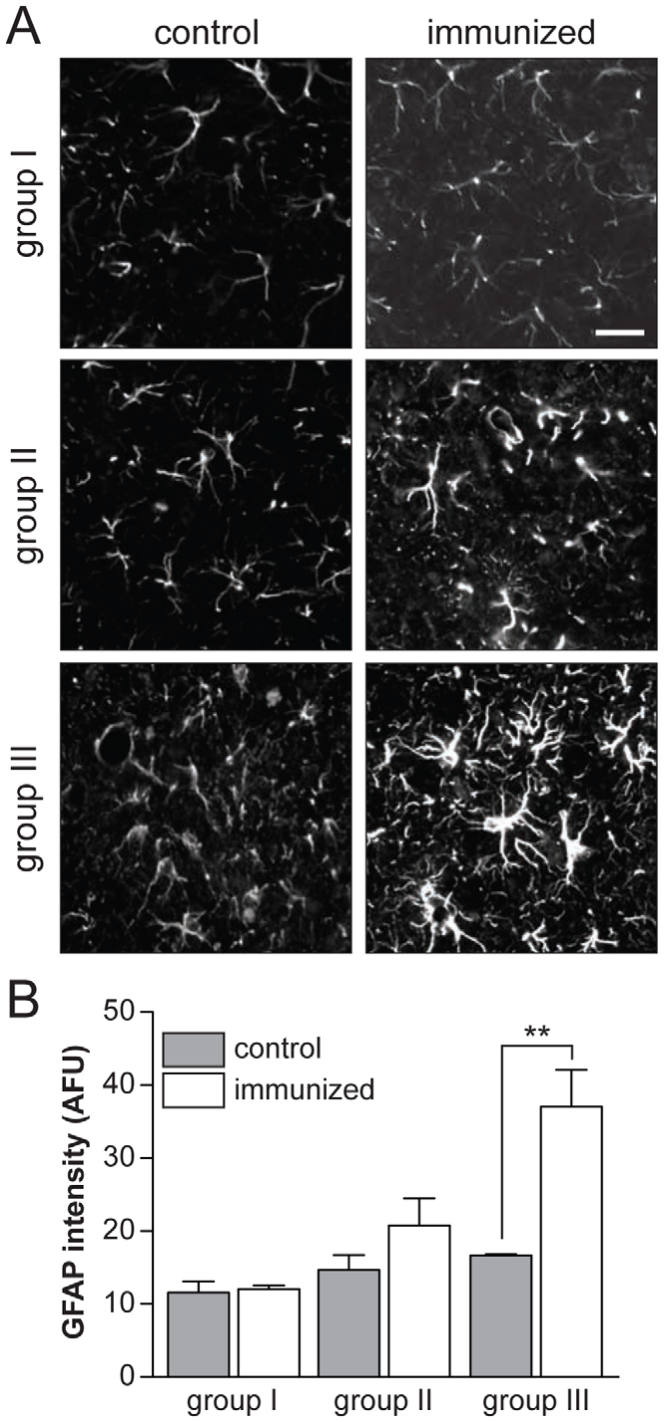
Tau-targeted immunization results in astrocytosis in old, but not young pR5 mice. (**A**) Immunohistochemistry with a GFAP-specific antibody stains astrocytes in brains of control and immunized 14 (group I), 17 (group II) and 24 (group III) months-old pR5 mice. Representative images of amygdalae show similar low GFAP staining of astrocytes in controls for all three groups, consistent with absence of an overt astrocytosis even in aged pR5 mice. In 14 months-old immunized pR5 mice (group I), GFAP staining is comparable to controls, while astrocytes stain more intensive in 17 months-old immunized pR5 (group II). In 24 months-old immunized mice (group III), GFAP staining is much stronger than in controls, and numerous astrocytes have adopted a tufted morphology, suggesting activation. Scale bar, 25 µm. (**B**) Quantification of GFAP staining intensity in the amygdala on serial sections of control and immunized pR5 mice revels a significant 2.2-fold increase in 24 months-old pR5 mice of group III compared to non-immunized pR5 controls (*p*<0.0001, n = 8).

Next, we tested if there is a cellular reaction associated with the decreased numbers of NFTs in antigen-treated pR5 mice. Interestingly, we found a strong activation of astrocytes in the amygdala of group III, as indicated by GFAP-positive tufted cells (**Fig. 4** **A**). Quantification of GFAP staining intensity revealed a significant 2.2-fold increase in antigen-treated pR5 mice of group III (*p*<0.001) compared to controls (**Fig. 4** **A and B**). Group II brains showed a trend towards astrocytosis, with some tufted cells. There was no change in astrocytic GFAP staining intensity in antigen-treated brains of group I pR5 mice, or controls of all age groups. Numbers of GFAP-positive cells appeared increased in control group III (**Fig. 4** **A and B**). Taken together, aged antigen-treated pR5 mice showed strong astrocyte activation in the amygdala.

## Discussion

In the present study, we showed that tau-targeted immunization slows the progression of tau pathology in P301L tau transgenic pR5 mice. Intraperitoneal injection of a peptide comprising the Ser396/Ser404 dual phospho-epitope of tau together with CFA or IFA, results in a persisting immune response that is associated with decreased tau phosphorylation, reduced numbers of NFTs and – in aged mice – astrocyte activation.

Tau-targeted immunization has been explored in different mouse models, including independent tau transgenic lines [14], [15], [16], [22]. Asumi *et al.* used a 30 amino acid peptide comprising the PHF-1 phospho-epitope to immunize 2 months-old JNPL3 mice, that express P301L mutant human tau in neurons and glia and are characterized by a rapid-progressive NFT pathology with an age of onset of 6.5 months [14]. In these mice, monthly immunization for up to 8 months reduced tau phosphorylation in brain and slowed the progression of tau-related motor deficits [14]. Therapeutic effects were less when mice were older at the start of treatment, indicating that some deficits cannot be restored [14]. Similar results were obtained by immunizing mice that express all human tau isoforms together with M146L mutant PS1 on a *Mapt^-/-^* background [15], however, an effect on NFT pathology in both models remains to be shown. Boimel *et al.* immunized 3 months-old K257T/P301S double mutant tau-expressing mice with a mixture of short peptides comprising the phosphorylation sites Ser202/Thr205, Thr212/Ser214 and Thr231, respectively [16]. By this approach, both tau phosphorylation and NFT burden was significantly reduced. This is consistent with our data of reduced NFT numbers in pR5, when treatment was initiated at 4 months of age, before the onset of NFT pathology. Together, both these previous and our own studies show that tau-targeted immunization with phosphorylated peptides reduces levels of tau phosphorylation and NFT burden in different mouse models of tauopathy, when treatment is started before or around the onset of NFT pathology.

However, it remained to be shown whether mice with advanced tau pathology benefit from tau-targeted immunization. Our study addressed this question by including mice with advanced NFT pathology, showing that even in very old mice, progression of the histopathological changes can be slowed by tau-targeted immunization. Having a total of three experimental age groups, our data suggests that while NFT numbers in our treatment groups I (4–16 mo) and II (8–17 mo) suggest that immunization would only slow the progression of the pathology, NFT numbers in group III (18–24 mo) were similar to untreated pR5 mice at 18 months of age, which is when treatment started. In line with a rather preventive than clearing effect on tau pathology in younger mice [14], our findings suggest that the treatment halts NFT formation in aged mice. Alternatively the rate of clearance of existing NFTs is equal to their *de novo* formation. However, due to the advanced age of the mice, we cannot exclude that the shorter treatment in group III has effects on NFT numbers. Overall, we showed that tau-targeted immunization improves NFT pathology of aged pR5 mice. Since tau-targeted immunization improved motor deficits in young JNPL3 mice together with reducing tau-phosphorylation but unchanged total tau levels [14], similar changes in tau phosphorylation in aged pR5 mice (our study) could also improve their deficits, although this remains to be shown.

Interestingly, we found a substantial activation of astrocytes in aged immunized pR5 mice (group III), as indicated by increased GFAP fluorescence intensity and morphological changes with tufted appearance. Hence, astrocytes may have a role in reducing tau pathology in aged pR5 mice. In the younger test groups I and II, however, there was no overt activation of astrocytes, although there was a trend of astrocyte activation in the intermediate age group II. This is consistent with a previous study, where immunization of 3 months-old K257T/P301S double mutant tau-expressing mice did not cause astrocyte activation [16]. These latter mice showed a slight increase in numbers of lectin-positive microglia, but they were not activated [16]. Taken together, to which degree the cellular response, and in particular the activation of astrocytes contributes to the effects of tau-targeted immunization remains to be shown. The differences in the cellular responses between the various studies may be explained by differences in immunization strategies (e.g. choice of peptides, target epitopes, and adjuvant) and mouse strains used.

There are a number of possible ways how tau-targeted immunization might slow disease progression. Firstly, antibodies that are capable of passing the blood brain barrier (BBB) (that is increasingly permeable with aging and in AD [23], [24]) and then enter neurons, modulating phosphorylation and/or degradation of tau directly. This concept remains controversial, since only one study revealed intraneuronal antibodies upon tau-targeted immunization [14], while another study showed antibodies in brain vessels, but not in neurons or brain parenchyma [16]. We did not see labeling of neurons with secondary anti-mouse antibodies in treated pR5 mice in the present study (data not shown). Interestingly, tau-specific antibodies were present in non-immunized pR5 mice, although titers were much lower than in immunized animals. Similarly, auto-antibodies were found in control JNPL3 and humanized tau mice [14], [15], however, their role remains unclear. Another possible mechanism is increased intracellular degradation of tau mediated by tau-targeted immunization. This may be supported by changes in cathepsin levels, which point to lysosome-mediated degradation in immunized K257T/P301S double mutant tau-expressing mice [16]. On the other hand, total tau levels remained unchanged upon immunization of JNPL3 mice [14], and staining with total tau antibodies was comparable in immunized and control pR5 mice (this study), arguing against overtly increased degradation of tau. It seems rather that tau-targeted immunization reduces the degree of tau phosphorylation in both young [14] and aged mice (our study), thereby possibly reducing soluble hyperphosphorylated tau species that are increasingly recognized as toxic [25]. Finally, tau-targeted immunization may clear specific tau species that are involved in intercellular spreading of tau pathology and inititiation of tau aggregation in recipient cells [25], [26]. This may involve clearing functions of astrocytes that we found to be activated in mice with higher NFT burden upon immunization. Taken together, the general mechanism(s) involved in the effects of tau-targeted immunization remain to be elucidated.

Immunization targeting proteins other than tau, which also form deposits in neurodegenerative disorders, has become an increasingly pursued therapeutic approach. The furthest advanced to date is Aβ-based active and passive immunization in AD. Despite some setbacks, different human trails have resulted in some promising results [13]. Furthermore, immunization against α-synuclein improved the pathology in a transgenic mouse model of Lewy body disease, a heterogeneous group of human disorders including Parkison's disease (PD) and dementia with Lewy bodies (DLB) [27]. Hence, vaccinating AD mouse models against tau fits well into a common treatment theme of tackling neurodegeneration. Being effective in mice, tau targeted immunization will need to proceed into human trials to proof efficacy.

## Materials and Methods

### Mice

All animal experiments have been approved by the Animal Ethics Committee of the University of Sydney (approval number K00/7-2009/3/5015). pR5 mice express P301L mutant human tau (2N4R) under control of the mouse Thy1.2 promoter in neurons [20]. Homozygous pR5 mice were used for this study. pR5 mice are maintained on a C57Bl/6 background, and wild-type controls were of the same genetic background.

### Immunization protocol

A peptide comprising twelve amino acids (KSPVVSGDTSPR) with serine (S) 396 and S404 being phosphorylated and linked to keyhole limpet hemocyanin (KLH) was used as antigen (OzPep, Australia) (**Fig. 1** **A**). The peptide was dissolved in phosphate-buffered saline (PBS) and mixed with either complete or incomplete Freund's adjuvant (CFA/IFA; Sigma, USA) at a ratio of 1∶1 (v:v) prior to intraperitoneal injection. Three groups of 4, 8 and 18 month old pR5 mice were injected with either 100 µg peptide-KLH or KLH (controls) with CFA, followed by injections with peptide-KLH or KLH in IFA two and four weeks later (**Fig. 1** **B**).

### Titer measurement

Four months after the initial immunization, blood was taken from the tail vein and serum isolated by centrifugation for antibody titer measurements using an enzyme-linked immunosorbant assay (ELISA). Briefly, 96-well Microlon plates (Greiner, Germany) were coated overnight with 10 µg/ml peptide in ELISA buffer (0.05% (v/v) Tween-20 (Sigma) in Tris-buffered saline (pH 7.5; Sigma)), washed and then blocked with 0.25% (w/v) bovine serum albumin (Sigma) in ELISA buffer for 30 minutes at room temperature. Serial dilutions of sera were incubated for 2 hours at room temperature and antigen-specific antibodies were detected with alkaline phosphatase (AP)-coupled anti-mouse antibodies (1∶15′000; Sigma) and ready-to-use pNPP dye (Sigma). The color reaction was measured at 405 nm in a Benchmark plus plate reader (Biorad). Titers are considered as the highest dilution with a positive assay signal.

### Histology

Six months after the initial immunization, mice were anesthetized and transcardially perfused with PBS followed by 4% paraformaldehyde (PFA) in PBS. Brains were extracted and fixed in 4% PFA/PBS over night at 4°C. Brains were then processed in a Excelsior tissue processor (Thermo, USA), embedded in paraffin and sectioned at 3 µm for immunohistochemistry (IHC) and 10 µm for Gallyas silver staining of NFTs. IHC and Gallyas staining have been described before [21]. Primary antibodies were against human tau (Tau-13, abcam), total tau (panTau, Dako), tau phosphorylated at Ser396/Ser404 (PHF-1, P. Davies) or Ser422 (PS422, Invitrogen) and glial fibrillary acidic protein (GFAP, Sigma). Alexa-labeled secondary antibodies to rabbit and mouse IgG were used for visualization (1∶250, Invitrogen).

### Quantification of histology

Gallyas-positive NFTs were counted on serial sections as previously described [18]. Briefly, NFTs in the amygdala were counted on five coronal sections of 50 µm distance per brain between the stereologic coordinates VD: −1.70 mm and −2.20 (in relation to the bregma).

Fluorescence intensity was determined as previously described [28], using the ImageJ software (NIH) and standardized pictures of the complete amygdala or CA1 region of the hippocampus from serial sections as outlined above.

### Statistics

Statistic analysis was done with the Prism 5.0 software (GarphPad) using Student's *t*-tests. All values are given as mean ± standard error of the mean.
